# Genome-wide association study of blood lipid levels in Southern Han Chinese adults with prediabetes

**DOI:** 10.3389/fendo.2023.1334893

**Published:** 2024-02-02

**Authors:** Zhenshu Gao, Changchun Pu, Leweihua Lin, Qianying Ou, Huibiao Quan

**Affiliations:** ^1^ Department of Endocrinology, Hainan Affiliated Hospital of Hainan Medical University, Hainan General Hospital, Haikou, China; ^2^ Department of Endocrinology, Hainan General Hospital, Haikou, China

**Keywords:** GWAS, prediabetes, HDL-C, TC, TG, LDL-C

## Abstract

**Background:**

Dyslipidemia is highly prevalent among individuals with prediabetes, further exacerbating their cardiovascular risk. However, the genetic determinants underlying diabetic dyslipidemia in Southern Han Chinese remain largely unexplored.

**Methods:**

We performed a genome-wide association study (GWAS) of blood lipid traits in 451 Southern Han Chinese adults with prediabetes. Fasting plasma lipids, including triglycerides (TG), total cholesterol (TC), high-density lipoprotein cholesterol (HDL-C) and low-density lipoprotein cholesterol (LDL-C) were assayed. Genotyping was conducted using the Precision Medicine Diversity Array and Gene Titan platform, followed by genotype imputation using IMPUTE2 with the 1000 Genomes Project (Phase 3, Southern Han Chinese) as reference. Single nucleotide polymorphisms (SNPs) associated with lipid levels were identified using mixed linear regression, with adjustment for covariates.

**Results:**

We identified 58, 215, 74 and 81 novel SNPs associated with TG, TC, HDL-C and LDL-C levels, respectively (*P* < 5×10^-5^). Several implicated loci were located in or near genes involved in lipid metabolism, including *SRD5A2*, *PCSK7, PITPNC1*, *IRX3*, *BPI*, and *LBP*. Pathway enrichment analysis highlighted lipid metabolism and insulin secretion.

**Conclusion:**

This first GWAS of dyslipidemia in Southern Han Chinese with prediabetes identified novel genetic variants associated with lipid traits. Our findings provide new insights into genetic mechanisms underlying heightened cardiovascular risk in the prediabetic stage. Functional characterization of implicated loci is warranted.

## Introduction

Diabetes mellitus is a prevalent chronic metabolic disease characterized by elevated levels of blood glucose (1). In 2021, there were 529 million people living with diabetes mellitus worldwide, which remains a substantial public health issue (2). The global burden of prediabetes is also substantial and growing (3). Prediabetes confers not only heightened risk for diabetes mellitus development, but also independent risk for cardiovascular disease (4, 5). Compared to healthy normoglycemic individuals, patients with prediabetes exhibit a greater propensity for atherogenic dyslipidemia, marked by elevated triglycerides (TG), total cholesterol (TC) and low-density lipoprotein cholesterol (LDL-C), alongside decreased high-density lipoprotein cholesterol (HDL-C) (6). This lipid profile significantly promotes atherosclerotic cardiovascular disease in the prediabetic population (7). Elucidating the pathogenic mechanisms underlying dyslipidemia in prediabetes is therefore imperative to inform strategies for complication prevention and management.

Lipid metabolism is a complex physiological process under polygenic regulation. While genome-wide association studies (GWASs) have uncovered several lipid-related loci in normoglycemic (8–11) and diabetes mellitus populations (12), focused genetic analyses in prediabetic cohorts remain scarce. Given the obvious dyslipidemia in prediabetes, studying the genetic characteristics of blood lipids in this population can reveal specific genetic variations. This can provide insights into disease susceptibility, aid risk stratification, and guide targeted interventions to mitigate cardiovascular risk.

In this study, we performed GWAS of lipid levels in 451 Han Chinese adults with prediabetes using the Precision Medicine Diversity Array. We aim to identify novel loci associated with TG, TC, HDL-C and LDL-C that may be specific to the prediabetic state. Significant variants will also undergo functional annotation. Our findings can facilitate personalized dyslipidemia management and cardiovascular risk assessment in this high-risk population.

## Materials and methods

### Study design and participants

**Figure 1:**
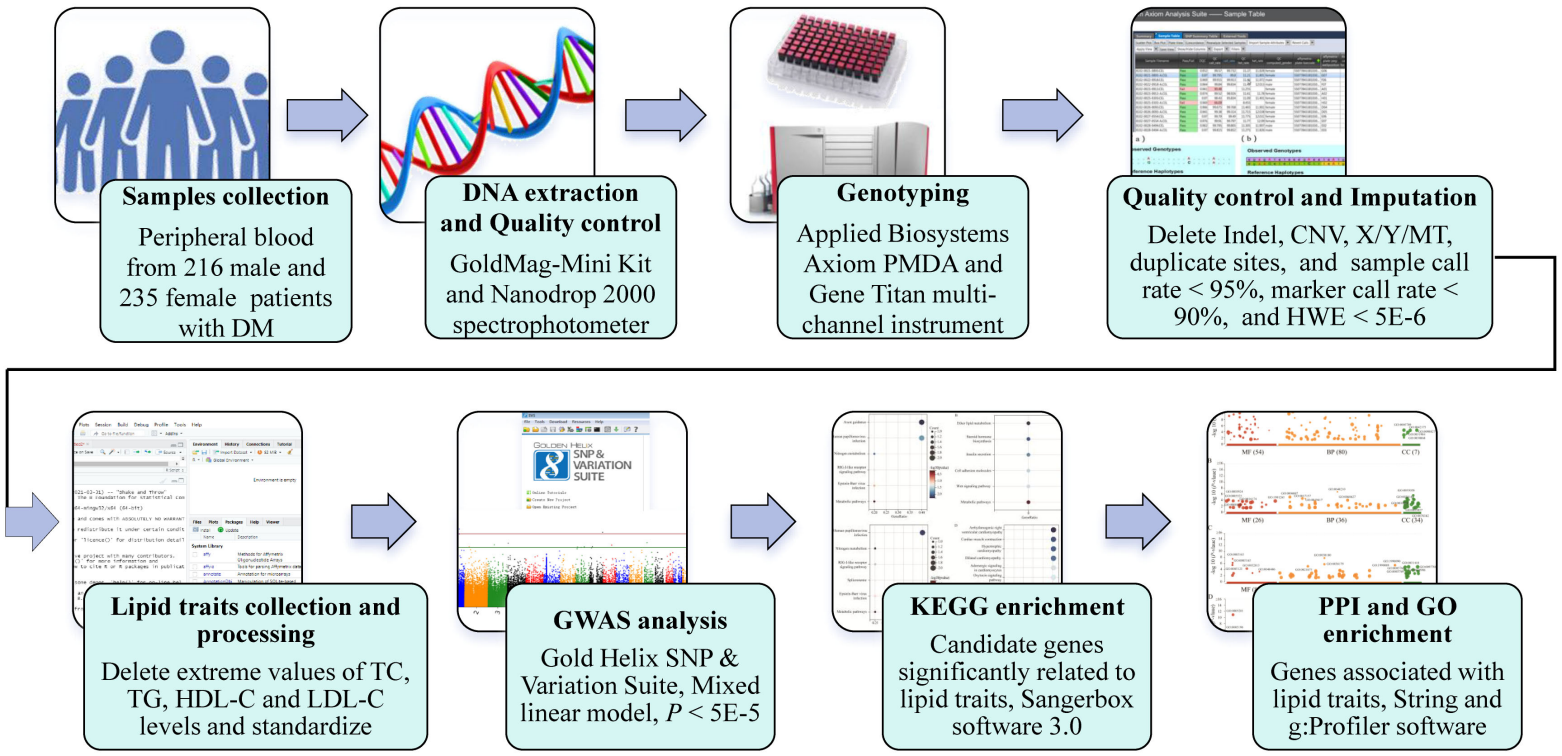
The flow chart of this study design.

To identify genetic loci associated with lipid traits, including TG, TC, HDL-C, and LDL-C, in the context of prediabetes among Southern Han Chinese, we conducted a GWAS (**Figure 1**). A total of 451 patients with prediabetes were recruited from the National Diabetes Prevalence Survey conducted by the Chinese Medical Association in 2017. Prediabetes was defined according to standard diagnostic criteria: 5.6mmol/L ≤ fasting plasma glucose (FPG) < 7.0mmol/L, or 7.8mmol/L ≤ 2-hours postprandial glucose (2hPG) < 11.1mmol/L, or 5.7% ≤ hemoglobin A1C (HbA1C) ≤ 6.4% (13, 14). All participants were at least 18 years old and had resided in the selected community for at least five years. Exclusion criteria were pregnancy, severe illness (e.g., cancer, kidney disease, acute infections), and cognitive impairment. Blood lipid levels were measured in a central laboratory using a Roche Modular autoanalyzer (Roche Diagnostics, Indianapolis, IN, USA).

This study was approved by the Ethical Committee of the Hainan Affiliated Hospital of Hainan Medical University (Med-Eth-Re (2019) 18) and adhered to ethical standards set forth by the committee and the Declaration of Helsinki. All participants provided written informed consent after being fully informed of the study’s purpose.

### DNA extraction and genotyping

Fasting peripheral blood (5mL) was collected from each participant in Ethylene diamine tetraacetic acid (EDTA) tubes and stored at −20°C. Genomic DNA was extracted from whole blood using the GoldMag-Mini Whole Blood Genomic DNA Purification Kit (GoldMag Co. Ltd., Xi’an, China) per manufacturer’s protocol. DNA concentration and purity were evaluated using the Nanodrop 2000 spectrophotometer (Thermo Fisher Scientific, Waltham, MA, USA). Samples were genotyped on the Applied Biosystems Axiom Precision Medicine Diversity Array (Thermo Fisher Scientific) using the GeneTitan platform. Genotyping results were managed and analyzed with Axiom Analysis Suite software (version 6.0). Of 108,825 genotyped markers, copy number variants, sex chromosome and mitochondrial SNPs, deletion and duplicated loci, and genotype calling rate < 95% and SNPs with HHardy-Weinberg equilibrium < 5×10^−6^ were excluded.

### Genotype imputation and quality control

Additional SNPs were imputed using IMPUTE2 software and the 1000 Genomes Project Phase III (Southern Han Chinese) reference panel, increasing genomic coverage to 9,378,219 SNPs. Post-imputation quality control removed non-biallelic variants, SNPs with imputation quality score ≤ 0.4, call rate < 90%, and HWE *p*-value < 5×10^−6^. The final imputed dataset contained 1,752,717 SNPs.

### Statistical analysis

Extreme outlier values for lipid traits were removed based on 3σ principle. Lipid indicators were then standardized using the RNOmni package in R software. Mixed linear regression analysis was performed to identify genetic variants associated with lipid traits (TG, TC, HDL-C, LDL-C) under an additive model with adjustment for age, gender, and smoking using Gold Helix SNP & Variation Suite software (version 8.7). Genome-wide significance was defined as *P* < 5 × 10^−5^. Pearson correlation analysis was conducted in SPSS 20.0 (IBM Corp, Armonk, NY) to assess associations between lipid indicators, with *p* < 0.05 considered statistically significant.

### Bioinformatics analysis

To explore the biological activities and functions of candidate lipid-related genes, the STRING database (https://cn.string-db.org/) was utilized to analyze protein-protein functional interactions. KEGG and Gene Ontology (GO) enrichment analyses were performed using SangerBox 3.0 (http://sangerbox.com/home.html) and g:Profiler databases (https://biit.cs.ut.ee/gprofiler/orth), respectively, to elucidate pathway and functional enrichments.

## Results

The baseline characteristics of the study participants are presented in **Table 1**. The mean age of prediabetic participants was 51.78 ± 14.19 years. The cohort comprised 235 females (52.1%) and 216 males (47.9%). Most were non-smokers (n = 341, 75.6%), with 28 occasional smokers (6.2%) and 82 regular smokers (18.2%). Mean lipid levels were as follows: TG 1.999 ± 1.075 mmol/L; TC 5.525 ± 1.096 mmol/L; HDL-C 1.438 ± 0.305 mmol/L; and LDL-C 3.052 ± 0.862 mmol/L. Significant positive correlations were observed between TC, LDL-C, and TG (**Supplementary Figure 1**).

**Table 1:** Basic characteristics of participants.

Variables		Participants (N = 451)
Age (years)	Mean ± SD	51.78 ± 14.19
Gender	Male	216 (47.9%)
	Female	235 (52.1%)
Smoking	No	341 (75.6%)
	Occasional	28 (6.2%)
	Regular	82 (18.2%)
TG (mmol/L)	Mean ± SD	1.999 ± 1.075
TC (mmol/L)	Mean ± SD	5.525 ± 1.096
HDL-C (mmol/L)	Mean ± SD	1.438 ± 0.305
LDL-C (mmol/L)	Mean ± SD	3.052 ± 0.862

TC, total cholesterol; TG, triglyceride; HDL-C, high-density lipoprotein-cholesterol; LDL-C, low density lipoprotein cholesterol; SD, standard deviation.

**Figure 2:**
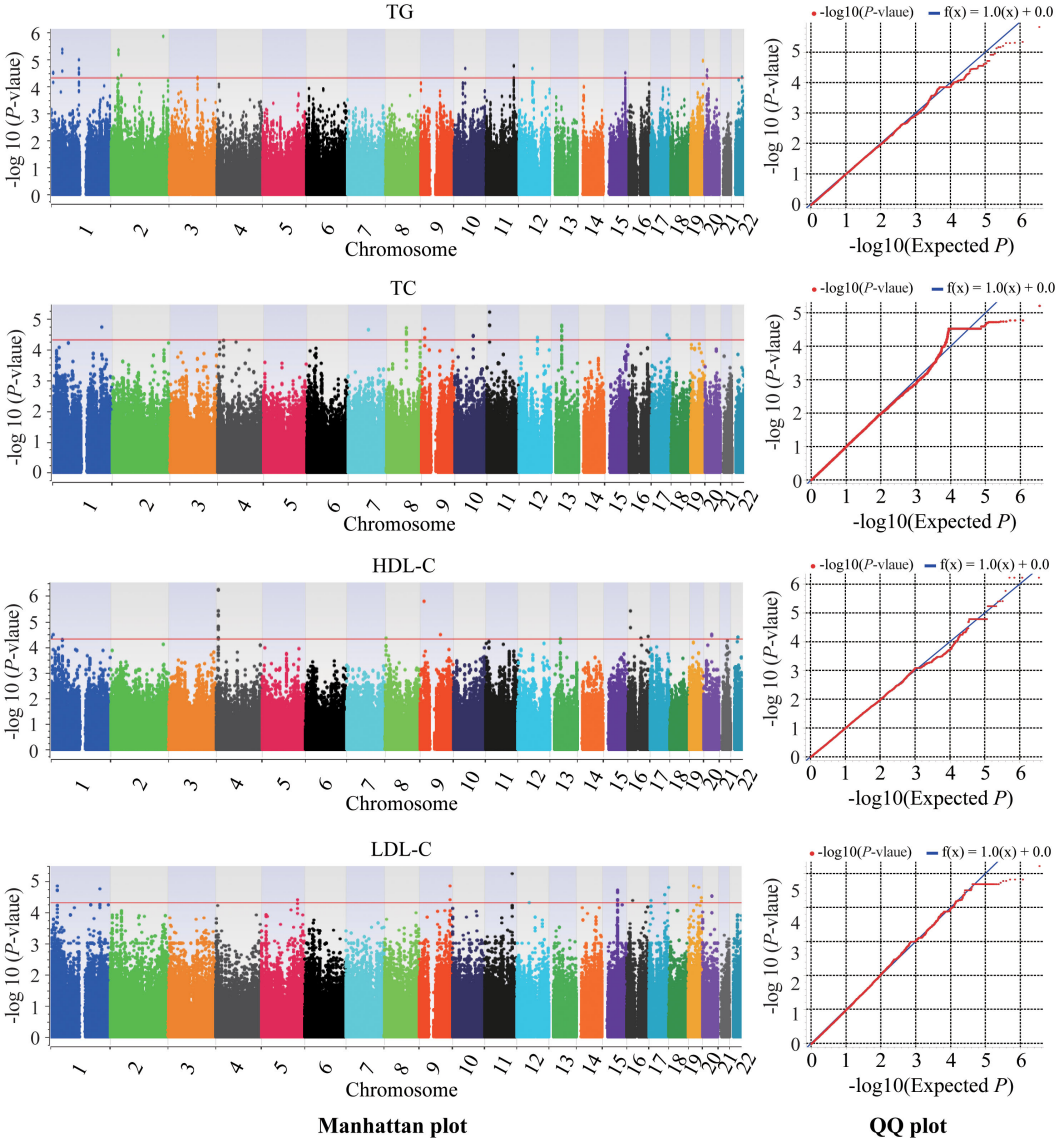
Manhattan plots and quantile-quantile plots of association of lipid traits in the GWAS. Chromosomes are shown on the x-axis, and the −log10 of the p-value on the y-axis. The red line represents the genome-wide significance cut off of 5 × 10^−5^. TC, total cholesterol; TG, triglyceride; HDL-C, high-density lipoprotein-cholesterol; LDL-C, low density lipoprotein cholesterol; QQ, quantile-quantile.

This genome-wide association study identified 58, 215, 74 and 81 novel SNPs surpassing genome-wide significance (*P* < 5×10^-5^) for association with TG, TC, HDL-C and LDL-C, respectively (**Supplementary Tables 1–4**; **Figure 2**). Lead SNPs were selected based on statistical significance and regional association patterns, resulting in 17 TG loci, 10 TC loci, 17 HDL-C loci, and 22 LDL-C loci (**Table 2**). Of these, 13 SNPs achieved *P* < 5×10^-6^, including rs12750886 near *TAL1* (*P* = 4.33×10^-6^), rs7598882 in *LINC01494* (*P* = 1.40 × 10^-6^), rs632148 (*P* = 4.59 × 10^-6^), rs534999 (*P* = 4.75 × 10^-6^), and rs558803 (*P* = 4.75 × 10^-6^) in *SRD5A2* associated with TG and rs62277650 (*P* = 3.86 × 10^-6^), rs76927959 (*P* = 3.86 × 10^-6^), rs62277655 (*P* = 5.85 × 10^-7^), rs7667521 (*P* = 5.85 × 10^-7^), rs7667528 (*P* = 5.85 × 10^-7^), and rs7697698 (*P* = 5.85 × 10^-7^) in *SORCS2*, rs914653 in *C9orf92*/*BNC2* (*P* = 1.69× 10^-6^), and rs56094199 in *LOC400499* (*P* = 3.88× 10^-6^) associated with HDL-C. Full lead SNP results are presented in **Table 2**.

**Table 2:** Lead SNPs associated with lipid traits in the GWAS.

Traits	SNP-ID	Chr	Allele		Functional	Genes	β	Beta SE	P-value
			Reference	Alternates					
TG	rs75060030	1:115632099	G	A	Intergenic	near VANGL1	0.620	0.139	1.05E-05
TG	rs12750886	1:47201578	C	T	Intergenic	near TAL1	0.495	0.106	4.33E-06
TG	rs12118524	1:8363969	G	A	Intron	*RERE*	1.357	0.323	3.29E-05
TG	rs7598882	2:218901233	C	T	Intron	*LINC01494*	0.329	0.067	1.40E-06
TG	rs632148	2:31580962	C	G	Intron	*SRD5A2*	-0.300	0.065	4.59E-06
TG	rs534999	2:31619435	C	T	Intron	*SRD5A2*	-0.306	0.066	4.75E-06
TG	rs558803	2:31619731	C	T	Intron	*SRD5A2*	-0.306	0.066	4.75E-06
TG	rs12486625	3:118869554	A	G	Intergenic	near IGSF11	-0.322	0.078	4.74E-05
TG	rs78286020	10:49337466	C	A	Intergenic	*C10orf71, DGEX*	-0.866	0.202	2.17E-05
TG	rs4938338	11:117102178	C	A	Intergenic	*SIK3, PAFAH1B2*	0.584	0.142	4.97E-05
TG	rs2306473	11:117227236	C	T	Synonymous	*PCSK7*	0.622	0.143	1.80E-05
TG	rs2277323	12:57615589	G	A	Synonymous	*ARHGEF25*	-0.446	0.104	2.27E-05
TG	rs12903987	15:89237741	A	C	Intergenic	*RLBP1, FANCI*	0.295	0.070	3.30E-05
TG	rs796769197	19:54233215	C	T	Intron	*LOC107985279*	0.943	0.212	1.13E-05
TG	rs6032829	20:10249643	C	T	Intron	*SNAP25*	0.438	0.105	3.94E-05
TG	rs7261869	20:13202311	G	A	Intron	*TASP1*	-0.334	0.079	2.63E-05
TG	rs5766271	22:44998703	C	G	Intron	*PHF21B*	0.692	0.169	4.78E-05
TC	rs147473747	1:208273372	G	A	Intergenic	near PLXNA2	-2.428	0.561	1.86E-05
TC	rs6468008	7:85015030	G	C	Intron	*SEMA3D*	-0.447	0.105	2.34E-05
TC	rs7009190	8:83592233	G	A	Intergenic	near RALYL	0.336	0.080	2.90E-05
TC	rs731691	9:14381256	A	G	Intron	*NFIB*	0.369	0.086	2.21E-05
TC	rs71479623	10:78410822	T	C	Intergenic	*LINC00856,LINC00595*	1.439	0.345	3.68E-05
TC	rs192936921	11:12278616	C	T	Intron	*MICAL2*	2.202	0.481	6.09E-06
TC	rs10859605	12:76301410	C	T	Intron	*LNCOG*	-0.277	0.067	4.24E-05
TC	rs7990302	13:44038059	A	G	Intergenic	*LACC1, SMIM2*	-0.327	0.076	1.81E-05
TC	rs2011941	17:67574759	C	T	Intron	*PITPNC1*	1.045	0.250	3.45E-05
TC	rs12451078	17:74096426	G	A	Intron	*LINC02074*	0.288	0.070	4.49E-05
HDL-C	rs12071900	1:8967281	G	A	Intron	*CA6*	-0.971	1.781	3.34E-05
HDL-C	rs111876969	1:989869	C	T	Intergenic	*HES4, ISG15*	-0.966	1.782	4.14E-05
HDL-C	rs62277650	4:7481619	C	T	Intron	*SORCS2*	-1.057	1.772	3.86E-06
HDL-C	rs76927959	4:7481774	A	G	Intron	*SORCS2*	-1.057	1.772	3.86E-06
HDL-C	rs62277655	4:7482841	G	T	Intron	*SORCS2*	-1.025	1.765	5.85E-07
HDL-C	rs7667521	4:7483240	G	A	Intron	*SORCS2*	-1.025	1.765	5.85E-07
HDL-C	rs7667528	4:7483259	G	A	Intron	*SORCS2*	-1.025	1.765	5.85E-07
HDL-C	rs7697698	4:7483276	T	C	Intron	*SORCS2*	-1.025	1.765	5.85E-07
HDL-C	rs78441207	8:4178216	A	C	Intron	*CSMD1*	-0.962	1.782	4.58E-05
HDL-C	rs914653	9:16332727	A	T	Intergenic	*C9orf92, BNC2*	-1.288	1.770	1.69E-06
HDL-C	rs112532863	9:86675781	T	C	Intron	*LINC02834*	-0.971	1.781	3.34E-05
HDL-C	rs56094199	16:11449409	G	T	Intron	*LOC400499*	-1.161	1.773	3.88E-06
HDL-C	rs9929651	16:54447680	C	T	Intergenic	*IRX3,CRNDE*	-1.023	1.782	4.81E-05
HDL-C	rs16974240	16:84596439	G	C	Intron	*COTL1*	-0.934	1.781	3.82E-05
HDL-C	rs78583633	20:32648618	T	G	3’UTR	*C20orf203*	-0.853	1.781	3.68E-05
HDL-C	rs551747680	22:30327021	C	A	Upstream	*TBC1D10A*	-0.936	1.782	4.23E-05
HDL-C	rs5753081	22:30344664	C	T	Intron	*SF3A1*	-0.936	1.782	4.23E-05
LDL-C	rs74875284	1:208286322	C	G	Intron	*LOC105372889*	-1.613	0.372	1.76E-05
LDL-C	rs144813326	1:30115489	G	A	Intergenic	*PTPRU, MATN1*	-0.955	0.218	1.44E-05
LDL-C	rs17112991	5:152286310	A	C	Intergenic	near NMUR2	0.906	0.218	3.91E-05
LDL-C	rs947510	9:130288831	C	T	Intron	*HMCN2*	-0.530	0.128	4.03E-05
LDL-C	rs145123934	11:116056778	A	T	Intron	*LOC105369513*	0.812	0.177	5.77E-06
LDL-C	rs58503378	12:52181240	C	T	Intron	*SCN8A*	0.796	0.194	4.95E-05
LDL-C	rs6494340	15:62662749	A	C	Intron	*TLN2*	-0.761	0.233	1.97E-05
LDL-C	rs34797393	16:24470341	T	C	Intron	*LOC105371143*	-0.629	0.152	4.33E-05
LDL-C	rs9912950	17:66938733	C	G	Intergenic	*CACNG4, CACNG5*	0.430	0.101	2.71E-05
LDL-C	rs117778326	17:82076110	C	T	Intergenic	*DUS1L, FASN*	-1.162	0.266	1.58E-05
LDL-C	rs56127676	17:9220790	G	A	Intron	*NTN1*	0.309	0.075	4.26E-05
LDL-C	rs141052786	19:22305219	A	C	Intron	*ZNF729*	-1.890	0.430	1.42E-05
LDL-C	rs7254892	19:44886339	G	A	Intron	*NECTIN2*	-0.504	0.115	1.57E-05
LDL-C	rs1126757	19:55368504	T	C	synonymous	*IL11*	0.327	0.079	4.55E-05
LDL-C	rs2305786	19:55379047	A	G	Downstream	*TMEM238*	0.348	0.083	3.43E-05
LDL-C	rs547687426	20:38320475	C	T	Intron	*BPI*	2.891	0.685	3.00E-05
LDL-C	rs186778434	20:38353785	C	T	Intron	*LBP*	2.891	0.685	3.00E-05
LDL-C	rs550319386	20:38426139	T	C	Upstream	*SNORA71B*	2.891	0.685	3.00E-05
LDL-C	rs183500202	20:38496106	C	T	Intron	*RALGAPB*	2.891	0.685	3.00E-05
LDL-C	rs186207347	20:38603823	G	A	Intron	*ARHGAP40*	2.891	0.685	3.00E-05
LDL-C	rs6028182	20:38893940	A	G	Intron	*PPP1R16B*	2.891	0.685	3.00E-05
LDL-C	rs13045621	20:39064094	C	T	Intergenic	near MAFB	2.891	0.685	3.00E-05

TC, total cholesterol; TG, triglyceride; HDL-C, high-density lipoprotein-cholesterol; LDL-C, low density lipoprotein cholesterol; Chr, chromosome; MAF, minor allele frequency; SE, standard error; G, guanine; C, cytosine; A, adenine; T, thymine.

Mixed linear regression analysis adjusted for age, gender, and smoking.

P < 5 × 10^−5^ indicates statistically significant.

**Figure 3:**
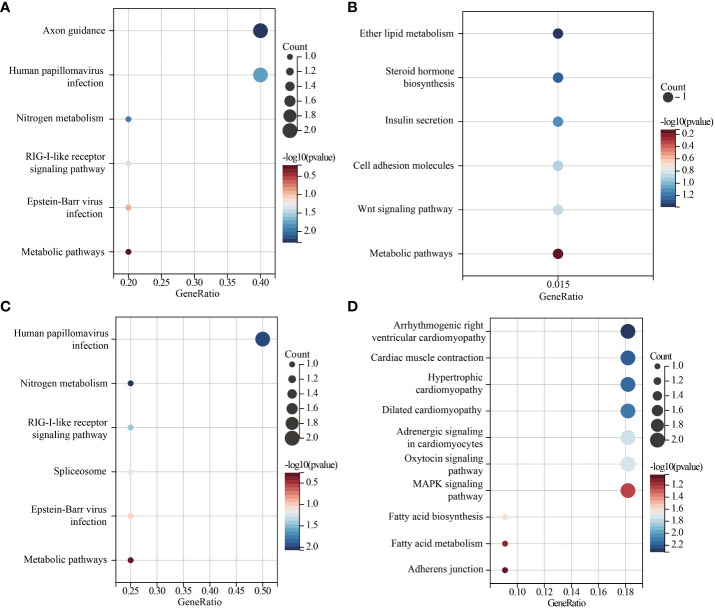
Scatter plot of KEGG enrichment of lead SNPs located or near genes. **(A)**. lead SNPs located or near genes associated with TG levels. **(B)**. lead SNPs located or near genes associated with TC levels. **(C)**. lead SNPs located or near genes associated with HDL-C levels. **(D)**. lead SNPs located or near genes associated with LDL-C levels. GeneRatio is the number of genes enriched in the KEGG entry divided by the total number of given genes.

**Figure 4:**
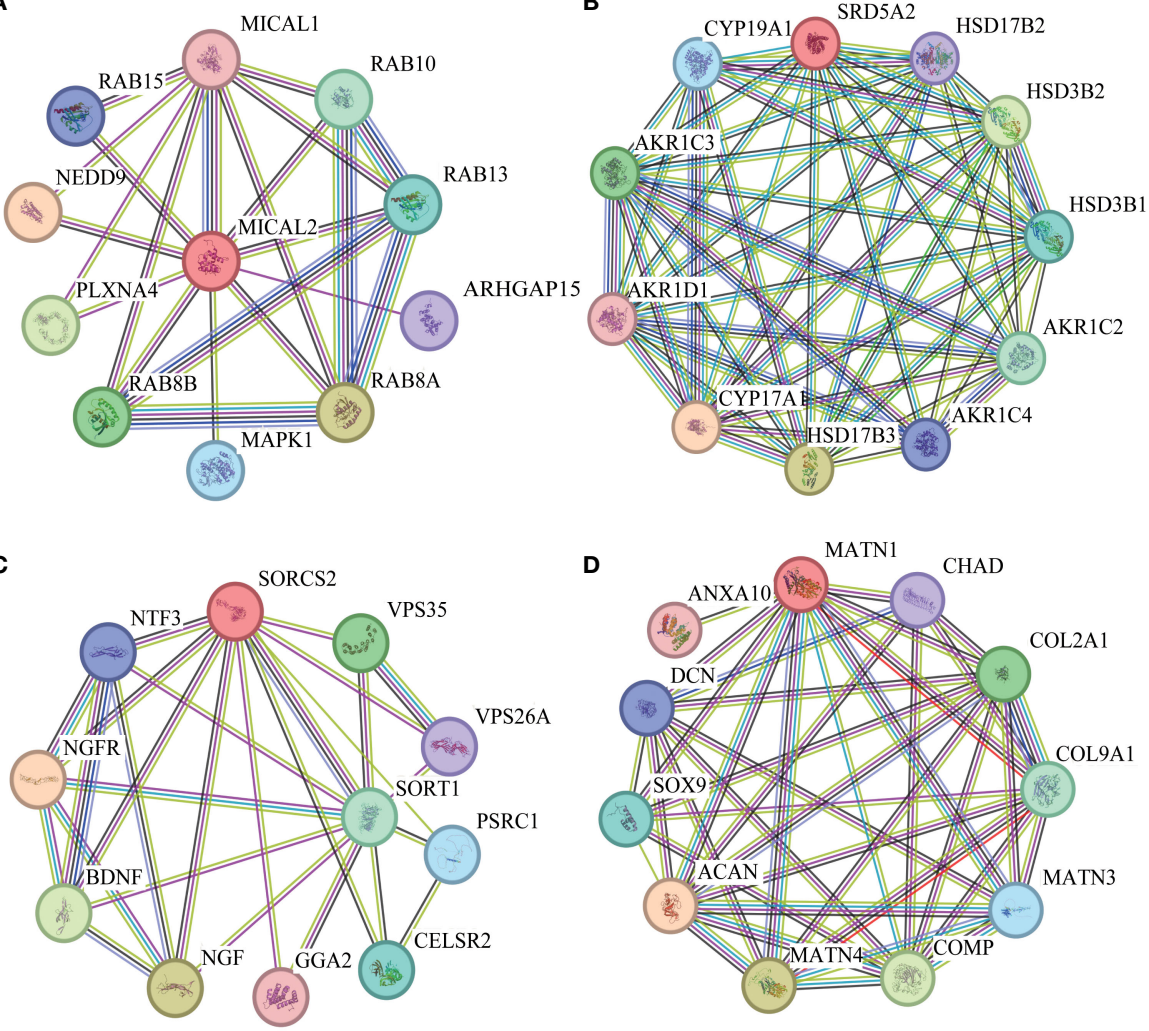
Protein-protein interaction (PPI) networks. **(A)**. PPI network diagram of interaction with MICAL2 protein. **(B)**. PPI network diagram of interaction with SRD5A2 protein. **(C)**. PPI network diagram of interaction with SORCS2 protein. **(D)**. PPI network diagram of interaction with MATN1 protein.

**Figure 5:**
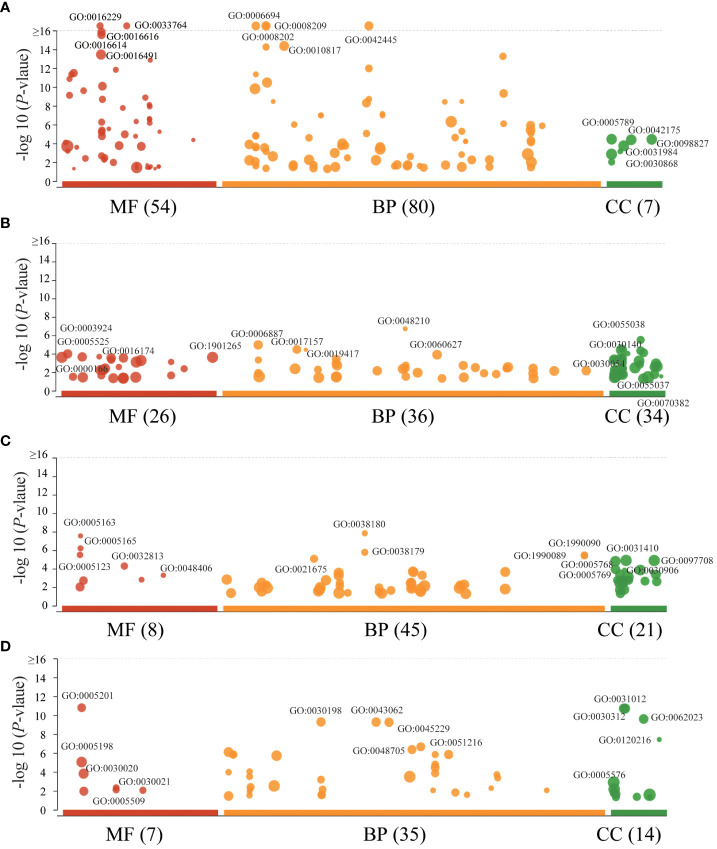
GO enrichment of interaction genes. **(A)**. GO enrichment of *MICAL2* interaction genes. **(B)**. GO enrichment of *SRD5A2* interaction genes. **(C)**. GO enrichment of *SORCS2* interaction genes. **(D)**. GO enrichment of *MATN1* interaction genes. The size of data points represents the number of enriched genes. BP, biological process; CC, cellular component; MF, molecular function.

The lead SNPs tagged distinct association signals in or near genes with known roles in lipid metabolism and transport, such as *SRD5A2*, *PCSK7*, *SNAP25*, *PITPNC1*, *IRX3*, *BPI*, and *LBP*. Meanwhile, KEGG enrichment analysis revealed that the genes mapped by the lead SNPs were enriched in several key pathways, including ther elipid metabolism, insulin secretion, Wnt signaling pathway, cardiac muscle contraction, MAPK signaling pathway, fatty acid metabolism, nitrogen metabolism, and metabolic pathways (**Figure 3**). In addition, we conducted interaction analysis and Gene Ontology (GO) enrichment analysis on the genes (*SRD5A2*, *MICAL2*, *SORCS2*, and *MATN1*) significantly associated with lipid traits in our GWAS results. The interaction analysis using the STRING database identified several potential protein-protein interactions among the lipid-associated genes (**Figure 4**). The GO enrichment analysis results revealed significant enrichment of several GO terms, includin gregulation of hormone levels, exocytosis, nerve growth factor signaling pathway, and extracellular matrix organization (**Figure 5**; **Supplementary Table 5**). In the next stage, we will follow up these SNPs in larger cohorts and perform functional characterization to elucidate the mechanisms underlying the genetic associations with lipid levels.

## Discussion

Our GWAS results identified numerous novel SNPs significantly associated with lipid traits, including 58 SNPs for TG, 215 SNPs for TC, 74 SNPs for HDL-C, and 81 SNPs for LDL-C, in a Chinese Han prediabetic poputation. Previous studies have shown that several lead SNPs located in or nearby genes, such as *SRD5A2*, *PCSK7*, *PITPNC1*, *IRX3*, *BPI*, and *LBP*, play a role in lipid metabolism (15–19). Meanwhile, enrichment analysis revealed that the lead SNPs located in or nearby genes were significantly enriched in elipid metabolism, insulin secretion pathways, and includin gregulation of hormone levels, exocytosis process.

Most individuals go through a prediabetes stage before progressing to full-blown diabetes. Recent research indicates that the long-term complications of diabetes manifest in individuals with prediabetes. These complications include cardiovascular disease, which is a leading cause of death in diabetes patients (20). It is well-established that dyslipidemia, including elevated TG levels and altered cholesterol profiles, plays a significant role in the development of cardiovascular disease. For instance, plasma TG levels of ≥2 mmol/L have been found to be associated with a two-fold increase in mortality from cardiovascular disease (21). In diabetic patients, elevated TC levels have been linked to an increased risk of cardiovascular disease (22). Increases in LDL-C levels have also been associated with a higher risk of myocardial infarction (23). Additionally, there is a U-shaped association between HDL-C levels and the risk of cardiovascular disease mortality, with a potential interaction between HDL-C levels and glycemic status (24). Therefore, by conducting a GWAS to identify the genetic variations associated with lipid levels in individuals with prediabetes, we can gain insights into the genetic basis of lipid metabolism in prediabetes patients.

Notably, our study identified six SNPs (rs10207755, rs522638, rs523349, rs632148, rs534999, and rs558803) in *SRD5A2* that showedassociations with TG levels in individuals with prediabetes. The *SRD5A2* gene encodes steroid 5-alpha reductase 2 and has been shown to suppress lipogenesis through inhibiting cortisol effects (15). The *SRD5A2* variant rs523349 was previously linked with a higher risk of the progression and death of patients with metastatic prostate cancer (25). Additionally, SNPs in the *SIK3*-*PAFAH1B2* intergenic region and *PCSK7*, along with rs6032829 in *SNAP25*, were associated with TG. These genes have demonstrated associations with TG levels in prior genome-wide studies in Korean population (26). Meanwhile, significant associations are detected between *SNAP25* polymprphism rs363050 and increasing fasting glucose, HbA1c, and lower insulin in type 2 diabetes mellitus (T2DM) patients (27). *WWOX* r517797882 was found to be significantly assciated with increased level of TG in T2DM in the Han Chinese population (28). However, it’s important to note that there is no overlap in the genomic locus examined between our study and the earlier investigation. This discrepancy may be attributed to the distinct focus of our study on prediabetes, whereas the previous research specifically addressed T2DM. The differences in the studied populations and conditions highlight the need for targeted investigations tailored to the specific stages and conditions within the spectrum of diabetes-related disorders.

We observed that the *PITPNC1* SNP rs2011941 was associated with TC levels in individuals with prediabetes. *PITPNC1*, a member of the phosphatidylinositol transfer protein family, promotes the thermogenes is of brown adipose tissue (17). In our study, we also identified an intergenic SNP (rs9929651) between *IRX3* and *CRNDE*, as well as multiple *SORCS2* SNPs, that showed significant associations with HDL-C levels in individuals with prediabetes. Previous studies have reported a negative correlation between *IRX3* expression levels of and serum TC, TG, and LDL-C levels (18). Additionally, *SORCS2* has been found to facilitate the release of endostatin from astrocytes and regulate post-stroke angiogenesis (29). Previous studuy also has observed that *PSMD6* rs831571 had a significant association with decreased HDL-C, while *WWOX* r517797882 significantly increased level of HDL-C in T2DM in the Han Chinese population (28). These findings highlight the importance of considering specific genes and pathways in the context of prediabetes and T2DM. However, it is important to note that additional studies are needed to validate these associations and elucidate the underlying mechanisms involved.

Futhermore, several SNPs were significantly associated with LDL-C levels, including rs7254892 in *NECTIN2*, rs1126757 in *IL11*, rs547687426 in *BPI*, rs186778434 in *LBP*, and rs13045621 near *MAFB*. These genes have established functional roles in regulating vascular inflammation, fibrosis, and lipid metabolism based on findings from recent studies. For example, recent studies have revealed pro-angiogenic, anti-apoptotic and anti-inflammatory properties of *NTN1* as well as preventing vascular dysfunction in diabetes (30). The NECTIN2 cell adhesion molecule has been shown to participate in lipid metabolism and serves as a potential biomarker for disease progression in carotid artery stenosis (31). Additionally, the cytokines *IL6* and *IL11* are upregulated under glucotoxic conditions, and *IL6*/*IL11*-mediated islet fibrosis may contribute to dysfunction in T2DM (32). Furthermore, the BPI-like proteins LBP and BPI are involved in lipid homeostasis and have been implicated in cardiovascular pathogenesis (19). However, the functional effects of many other lead SNPs located in or nearby novel candidate genes still need to be explored and validated experimentally. Elucidating the mechanisms by which these uncharacterized genes influence lipid traits will provide new insights into dyslipidemia in the prediabetic state.

In our study, we found that genes significantly linked to lipid levels in prediabetes are involved in regulating various pathways, including ether lipid metabolism, steroid hormone biosynthesis, cell adhesion molecules, Wnt signaling pathway, fatty acid biosynthesis, MAPK signaling pathway, fatty acid metabolism, and metabolic pathways. Additionally, our GO enrichment analysis showed that proteins interacting with SRD5A2, MICAL1, and MATN1 proteins may be engaged in hormone and steroid metabolism, regulation of exocytosis and vesicle-mediated transport, and extracellular structure organization, respectively. These discoveries strongly indicate that the genes associated with these pathways play a critical role in regulating lipid levels in individuals with prediabetes. Delving deeper into the intricate mechanisms governing these pathways will help uncover potential therapeutic targets and elucidate the molecular mechanisms underlying disease development. This provides valuable insights for further research into the molecular regulation of prediabetes, enriching our overall understanding of the associated biological processes.

This study has several limitations. First, the sample size was small with only 400 patients, which reduces power to detect SNPs with minor effects. Expanding the cohort would improve statistical power. Second, only Southern Han Chinese were included, limiting generalizability to all Chinese prediabetics. Multi-center studies across diverse populations are needed. Third, environmental and lifestyle factors were not well controlled, which could influence blood lipids. Detailed lifestyle data should be collected in future studies and considering gene-gene and gene-environment interactions. Fourth, as discovery research, only preliminary SNP screening was done without functional validation of novel hits. Experimental studies are needed to verify biological roles. Fifth, this study did not identify SNPs related to dyslipidemia in prediabetes. Further research will consider stratified patients in prediabetes according to lipid levels to identify SNPs related to dyslipidemia in prediabetes. Finally, comparison with healthy controls was lacking, thus it remains unclear if identified SNPs are specific to prediabetes. Comparisons to normoglycemic cohorts would aid in elucidating pathogenic mechanisms underlying dyslipidemia. Addressing these limitations in follow-up studies will provide more robust insights into the genetic architecture of lipid abnormalities in prediabetes.

## Conclusions

This GWAS provides confirmatory evidence for associations of multiple lipid loci (58 SNPs for TG, 215 SNPs for TC, 74 SNPs for HDL-C, and 81 SNPs for LDL-C) in a prediabetic Southern Chinese Han population, while also uncovering novel SNP associations warranting further functional characterization. The findings yield insights into dyslipidemia genetic mechanisms in prediabetes.

## Data availability statement

The data presented in this study are deposited in the Zenodo repository, accessible via the DOI number 10.5281/zenodo.10586090.

## Ethics statement

The studies involving humans were approved by Hainan Affiliated Hospital of Hainan Medical University. The studies were conducted in accordance with the local legislation and institutional requirements. The participants provided their written informed consent to participate in this study. Written informed consent was obtained from the individual(s) for the publication of any potentially identifiable images or data included in this article.

## Author contributions

HQ: Conceptualization, Project administration, Writing – original draft. ZG: Investigation, Writing – original draft. CP: Data curation, Writing – review & editing. LL: Formal analysis, Methodology, Writing – review & editing. QO: Conceptualization, Methodology, Project administration, Writing – review & editing.
