# Supplementary figures and images for: Genome-wide association study of blood lipid levels in Southern Han Chinese adults with prediabetes
Source: Front Endocrinol (Lausanne). 2024 Feb 2;14:1334893. doi: 10.3389/fendo.2023.1334893 (PMC10869499; doi:10.3389/fendo.2023.1334893)

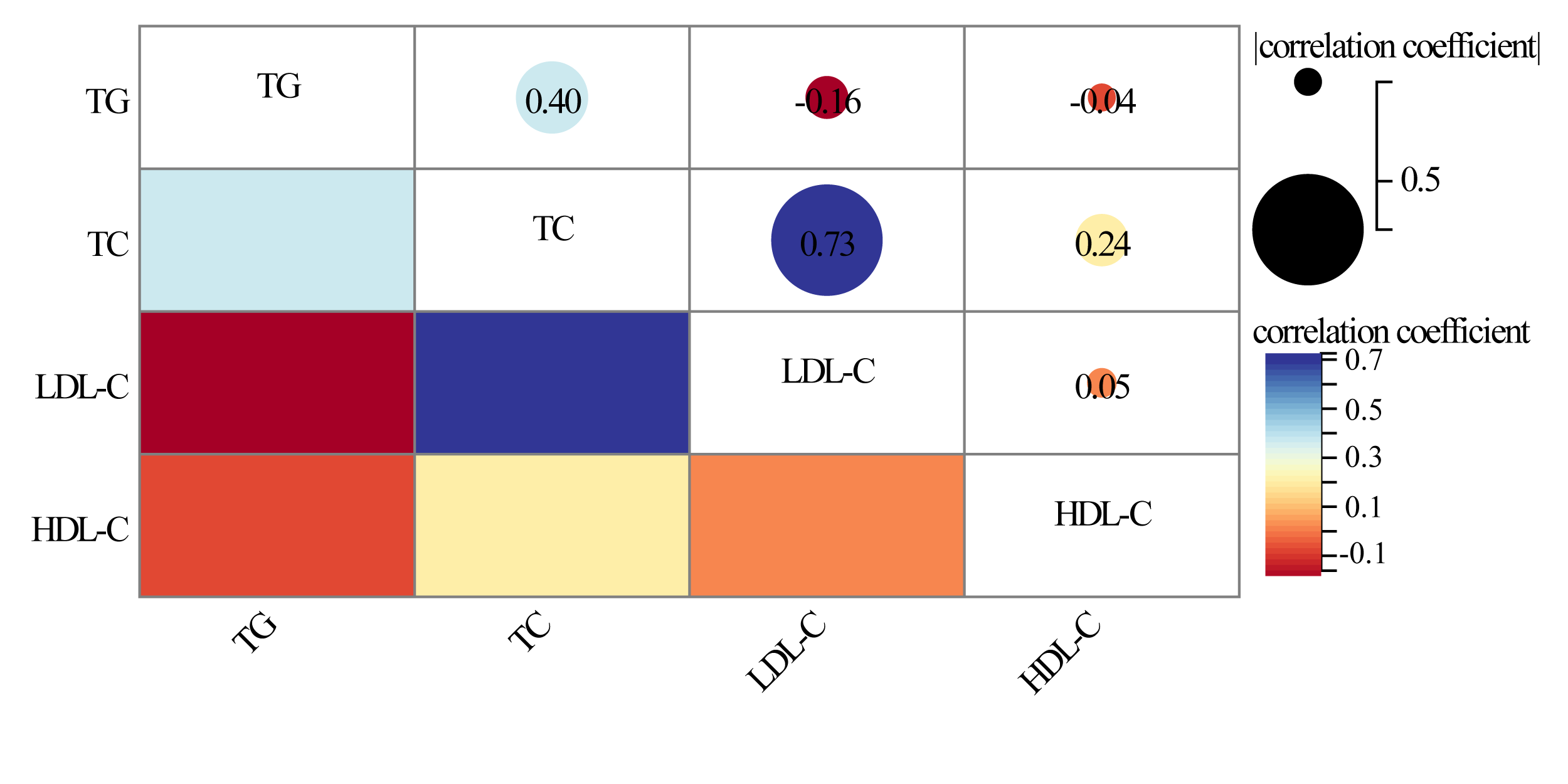

Supplement: Supplementary file 1 [file Image_1.tif]
